# Common Complications of Cesarean Section During the Year 2017 in King Abdulaziz Medical City, Jeddah, Saudi Arabia

**DOI:** 10.7759/cureus.12840

**Published:** 2021-01-21

**Authors:** Aljoharah A Aljohani, Hatim M Al-Jifree, Refal H Jamjoom, Rawan S Albalawi, Amal M Alosaimi

**Affiliations:** 1 Medicine, King Abdullah International Medical Research Center, Jeddah, SAU; 2 Medicine, King Saud Bin Abdulaziz University for Health Sciences College of Medicine, Jeddah, SAU; 3 ‏Gynecological Oncology, King Saud Bin Abdulaziz University for Health Sciences, Jeddah, SAU; 4 Oncology, King Abdullah International Medical Research Center, Jeddah, SAU; 5 Oncology, Ministry of National Guard Health Affairs, Jeddah, SAU; 6 Obstetrics and Gynecology, King Abdullah International Medical Research Center, Jeddah, SAU; 7 Obstetrics and Gynecology, King Saud Bin Abdulaziz University for Health Sciences College of Medicine, Jeddah, SAU; 8 Oncology, College of Medicine, King Saud Bin Abdulaziz University for Health Sciences, King Abdullah International Medical Research Center, Jeddah, SAU

**Keywords:** cesarean section, complications, cesarean delivery, c section, maternal complications, cs, fetal complications, multiparity, emergency and elective cesarean, pregnancy

## Abstract

Cesarean section (CS) is one of the most well-known major obstetrics surgeries and one of the oldest operations in the area of abdominal surgery. It is used for the purpose of delivering the newborn and the placenta through the abdominal wall incision (laparotomy) as well as Uterine incision (hysterotomy), followed by suture of the uterus and abdominal wall layers. Most common maternal complications internationally, according to literature were bleeding and wound infection. Most common fetal complications according to the literature were depressed Appearance, Pulse, Grimace, Activity, and Respiration (APGAR) score, low blood pH and ICU admissions. The aim of this study is to determine the most common CS complications among all the deliveries at King Abdulaziz Medical City (KAMC) in Jeddah in 2017 and to estimate maternal and fetal complication rates following CS. Comparing the rate of complication between residents and non-residents physicians performing a CS was tested as a secondary outcome. To the best of our knowledge, this is a novel research in this medical center at KAMC Jeddah that will aid in quality improvement in both clinical services and training activities of residents. The maternal and fetal complication rates were assessed in a cross-sectional chart review study. In order for this method to be achieved, a secondary data collection sheet was constructed to collect all eligible patient health records. This literature review was based on estimating the rate of CS complications for the mothers and their neonates that founded approximately 7% and 6%, respectively, where the procedure was either performed electively or emergently within the period of 1 January to 31 December 2017. Also, all patients with medical and surgical conditions were included while intrauterine fetal death was excluded. The most common maternal complications documented in our population were bleeding and wound extension, while the most common fetal complications were low APGAR score and NICU admissions. No statistical significance was found in either complications in terms of the correlation between demographical factors, maternal health conditions and gravida status. As for the secondary objective, the association between operator level of training and rate of complications revealed a higher percentage rate of maternal and fetal complications among consultants, which were 6.2% and 8.2%, respectively, mainly because the number and complexity of their cases in comparison to cases held by residents and others.

## Introduction

Cesarean section (CS) is one of the most well-known major obstetrics surgeries and also, one of the oldest operations in the area of abdominal surgery. It is used for the purpose of delivering the newborn and the placenta through the abdominal wall incision (laparotomy) as well as Uterine incision (hysterotomy ), followed by suture of the uterus and abdominal wall layers [1]. World Health Organization (WHO) had stated based on the international health care community report, that the acceptable rate for CSs should be within the range of 10% to 15%. Ever since the number of CSs have increased in both non-developed and developed countries. WHO also reported an extensive variety of CS delivery rate that was accounted across the world [2]. A secondary analysis of two WHO multi-country surveys which was conducted by Joshua P, et al. found a clear evidence of CS that increased from 26.4% to 31.2%, in many countries. These countries were Argentina, Brazil, Cambodia, China, the Democratic Republic of the Congo, Ecuador, India, Japan, Kenya, Mexico, Nepal, Nicaragua, Niger, Nigeria, Paraguay, Peru, Philippines, Sri Lanka, Thailand, Uganda, and Vietnam [3]. Furthermore, in Europe, the rates differ considerably, with the estimated rates of 15% in Norway and the Netherlands, around 17% in Sweden and Finland, and 37.8% in Italy [4].

In the Kingdom of Saudi Arabia, the cesarean delivery rate accounts for approximately 10% of all births, increasing up to 20% in tertiary centers [5]. While in Saudi Arabia, the Ministry of Health reported that the rate of CS had been indicated to be the second most commonly performed surgical procedures in Saudi Arabia, due to both medical and elective reasons [6].

The total number of operations performed in the Ministry of Health Hospitals in 2015 was 504,234, in which, CS deliveries constituted 20.9% of the total deliveries. Also, Gynecology and Obstetrics operations were in the second place by 23% after the general surgery constituted which had the highest percentage 24% [6]. In the last 20 years, a steady increase in CS rate was conducted at King Abdulaziz Medical City (KAMC), Riyadh, Saudi Arabia. In fact, CS rate has increased from 8% to 21% between the years 1993 and 2013 [7]. CS constitutes a notable surgical technique and, accordingly, is connected with various surgical complications [8]. Given the global increase in the rate of cesarean deliveries, with the associated higher morbidity and mortality [9].

Maternal complications of CS can be demonstrated by short- and long-term complications. Starting with the short-term postoperative complications, bleeding and wound infections are the most common, earliest and the most critical considerable surgical complications may lead to a prolonged hospitalization [1]. Moreover, pain and postoperative infections (in 3%-15% of patients) [1] such as urinary tract injury, laceration cellulitis, pelvic cellulitis and endometritis are all considered early postoperative complications. Furthermore, CS long-term postoperative complications such as wound subcutaneous abscess was noticed to be appeared approximately 22 days after surgery [10]. Other complications are known to take some time to develop such as pelvic abscess, thromboembolic complications, deep venous thrombosis (DVT), which has an incidence of three to five times to happen after CS than after vaginal delivery [11]. The risk of DVT is that it can progress to pulmonary embolism if untreated. It typically presents as unilateral leg tenderness, swelling, and a palpable cord [11]. Some studies regarding long-term complications of CS indicated that mothers who had their first born delivery by CS are at high risk of having placental abruption and placenta previa in the following pregnancy by 30% and 40%, respectively, contrasted with first birth by the vaginal delivery [12].

Fetal complications are also reported when CS is performed, infants delivered by elective CS delivery have an immense extended threat of transient tachypnea in the infant and pulmonary hypertension when contrasted and those conceived vaginally. Utilized fetal ventilator support is needed as a marker of presumed pulmonic ailment to assess the part of work in lung liquid discharge. There was a measurably critical contrast in fetal ventilator use between all patients with a previous CS compared to those with previous vaginal delivery [13]. Prolonged hospitalization for more than seven days was reported for infants born to mothers with a history of a prior CS birth. Babies who were delivered by CS had shown an increased risk for having Type 1 Diabetes Mellitus by two-fold compared to babies delivered vaginally [8,14]. Depressed Appearance, Pulse, Grimace, Activity, and Respiration (APGAR) score, low blood PH, ICU admission are some of the fetal complications. Other possible complications can be triggered as well [1,15]. CS rates varied based on some factors, such as variations in regions, financial status, countries, and women preferences. The rate also varies depending on the type of medical practice. Also, a high increase is observed when pregnant females are managed through private practice in comparison to public practice [8]. This literature review focuses on the rate variation of CS complications based on the medical practice of resident and non-resident. To the best of our knowledge, this is a novel research in this medical center at KAMC Jeddah that will aid in quality improvement in both clinical services and training activities of residents. Furthermore, our population is different from any other. Usually, they have multiparty, so we want to know if this type of population has a specific or higher type of complications in comparison to other populations.

## Materials and methods

The method used in this research was cross-sectional retrospective chart review study. A secondary data collection sheet was constructed and all eligible health record files of patients were reviewed by the investigators in this research. Furthermore, all information were gathered in a confidential anonymous way in the secondary data collection sheets for analysis. Descriptive statistics were presented as the mean and standard deviation (Table 1). The number of women whose data were collected and underwent CS was 281. Demographic features such as BMI, age, and nationality were collected and reported as 29.060 Mean for the BMI and SD of 6.11204. Age had a mean of 30.65 and an SD of 5.884. As for the nationality, 97.2% were Saudi, with 2.8% being non-Saudi such as Yemeni, Syrian, Pakistani, Philippino, and others. Variable study factors such as smoking status, diabetes, and hypertension prevalence were counted as well. Outcome factors were documented which were any complications, maternal and fetal.

 

**Table 1:** Demographic characteristics of all participants (n = 281). CS: cesarean section.

Characteristics	Value
Age of mothers (years)	30.65±5.88 (17.00-46.00)
BMI (kg/m^2^)	29.06±6.11 (17.00-47.00)
Nationality	
Saudi	273 (97.2%)
Non-Saudi	8 (2.8%)
Number of total pregnancies	3.26±2.30 (1.00-13.00)
Number of past deliveries	1.81±1.82 (0.00-8.00)
Number of term deliveries	0.03±0.26 (0.00-3.00)
Number of abortions	0.43±0.88 (0.00-6.00)
Past deliveries	1.84±1.83 (0.00-8.00)
Past CS	1.01±1.16 (0.00-5.00)
Comorbidity	
No	168 (59.8%)
Yes	113 (40.2%)
Gestational diabetes mellitus	51 (18.1%)
Hypothyroidism	24 (8.5%)
Diabetes mellitus	18 (6.4%)
Hypertension	10 (3.6%)
Asthma	10 (3.6%)
Preeclampsia	7 (2.5%)
Pregnancy cholestasis	4 (1.4%)
Anemia	4 (1.4%)
Others	27 (9.6%)

## Results

Within the period of 1 January to 31 December 2017, number of CS Complications in KAMC-J was estimated for all women who underwent CS whether the procedure was elective or emergency, and whether the gestational status was full-term or pre-term. Also, all patients with medical and surgical conditions were included. We excluded intrauterine fetal death. Out of 281, approximately 7% suffered from maternal complications with the percentage of bleeding 2.1%, wound extension 2.1% (Table 2). Uterine atony and urinary bladder injury were accounted for 1.1% and 0.7%, respectively. Other maternal complications such as cyst rupture, uterine artery injury, hematoma formation, and others were estimated to be 0.7%.

**Table 2:** Cesarean section types and their complications of all participants (n = 281). NICU: neonatal intensive care unit; CS: cesarean section; APGAR: Appearance, Pulse, Grimace, Activity, and Respiration.

Characteristics	Values
Type of CS	
Elective	99 (35.2%)
Emergency	182 (64.8%)
Operators	
Consultant	195 (69.4%)
Associate Consultant	13 (4.6%)
Assistant Consultant	10 (3.6%)
Staff Physician	2 (0.7%)
Resident	27 (9.6%)
Others	34 (12.1%)
Complications	
No	262 (93.2%)
Yes	19 (6.8%)
Maternal complications	
No	262 (93.2%)
Yes	19 (6.8%)
Bleeding	6 (2.1%)
Wound extension	6 (2.1%)
Uterine atony	3 (1.1%)
Urinary tract injury	2 (0.7%)
Others	2 (0.7%)
Fetal complications	
No	265 (94.3%)
Yes	16 (5.7%)
NICU admission	6 (2.1%)
Low APGAR score	6 (2.1%)
Fetal stress	2 (0.7%)
Fetal death	2 (0.7%)
Amount of blood loss (ml)	774.12±178.74 (100.00-2000.00)
Number of patients received blood transfusions	21 (7.5%)

Fetal complications added up to approximately 6% with NICU admissions percentage of 2.1% and low APGAR score of 2.1% (Table 2). Fetal death and fetal distress were extremely rare and reported as 0.7%.

Association of primigravida vs multigravida mothers and rate of maternal complications showed no significance, but with a slight increase of risk in multigravida mothers, 5.9% vs 7.0% (Table 3).

 

**Table 3:** Gravida and rate of maternal complication association.

Variables	Rate of complications		Total
	No	Yes	
Gravida Primigravida Count % within Gravida	64 (94.1%)	4 (5.9%)	68 (100.0%)
Gravida Multigravida Count % within Gravida	198 (93.0%)	15 (7.0%)	213 (100.0%)
Total Count % within Gravida	262 (93.2%)	19 (6.8%)	281 (100.0%)

The percentage of emergency CS was 64.8% and 35.2% for elective CS. The association was tested between the two deliveries, elective and emergency CS, as well as the rate of maternal complication (Table 4). There was a slight increase in the risk of maternal complications in elective CS but no significance, 6.6% for emergency CS vs 7.1% for elective CS. As for the association between emergency CS and elective CS with fetal complications, emergency CS reported a total of 8.2% fetal complications vs elective CS with 1% (Table 4). The commonest health condition reported among our population was GDM/DM with a percentage of 24.6%. Testing the association between GDM/DM and rate of maternal complications was not significant. Association between the number of past CS and maternal complications was not significant as well as fetal complications. 

 

**Table 4:** Cross-tabulation between fetal and maternal complications and types of CS. CS: cesarean section.

Variables	Maternal complications		Fetal complications	
	No (n = 262)	Yes (n = 19)	No (n = 265)	Yes (n =16)
CS types				
Elective CS (n = 99)	92 (92.9%)	7 (7.1%)	98 (99.0%)	1 (1.0%)
Emergency CS (n = 182)	170 (93.4%)	12 (6.6%)	167 (91.8%)	15 (8.2%)
Past CS				
No (n = 122)	114 (93.4%)	8 (6.6%)	115 (94.3%)	7 (5.7%)
Yes (n = 159)	148 (93.1%)	11 (6.9%)	150 (94.3%)	9 (5.7%)

Looking into the association between rate of complications and level of operators (Consultant vs Residents and others) was tested using Chi-square test, details are mentioned in the discussion.

## Discussion

**Figure 1:**
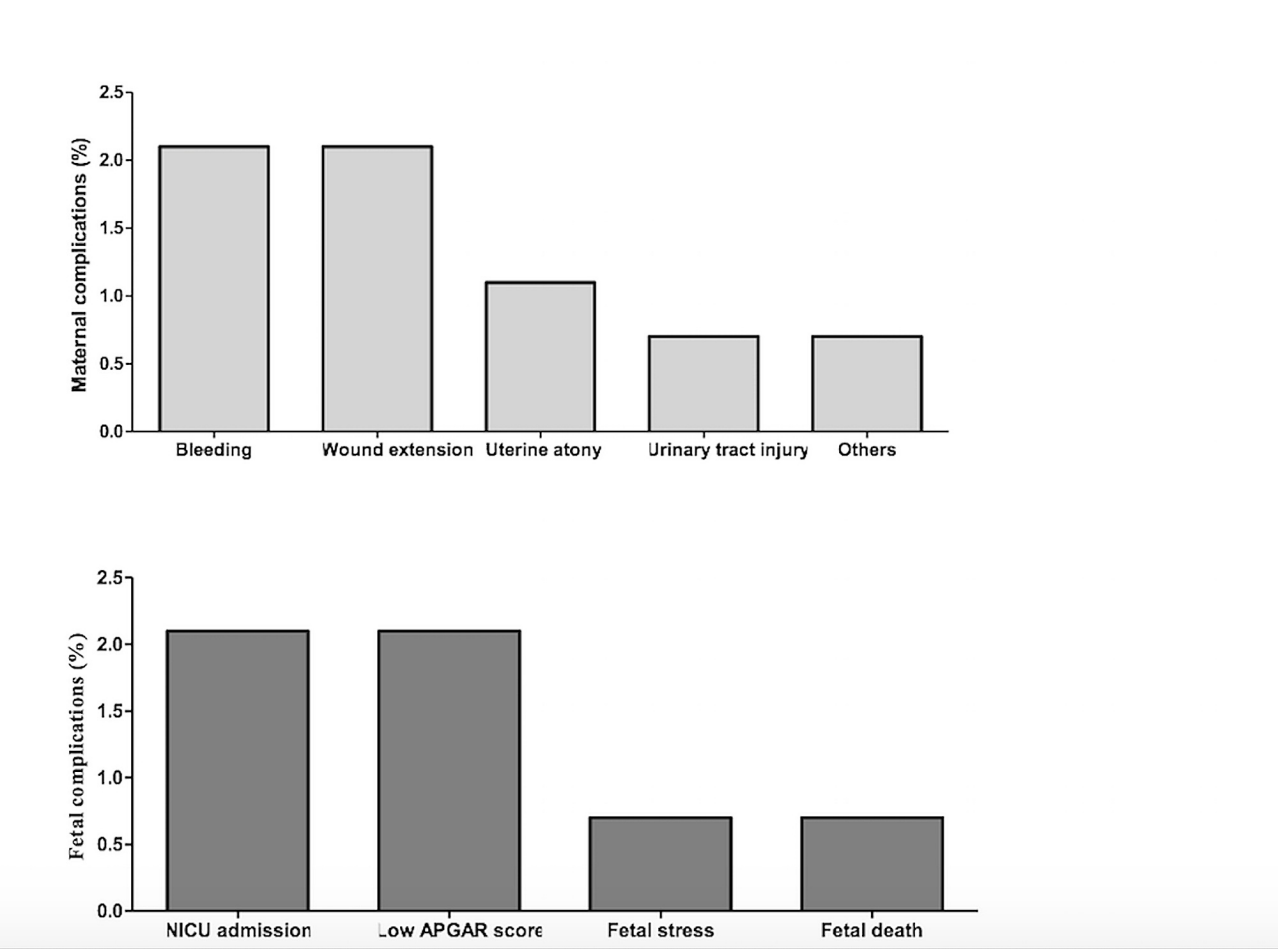
Maternal and fetal complication CS rate among KAMCJ in the year of 2017. CS: cesarean section; KAMC-J: King Abdulaziz Medical City, Jeddah; NICU: neonatal intensive care unit; APGAR: Appearance, Pulse, Grimace, Activity, and Respiration.

The purpose of this literature review was to estimate the number of CS complications in KAMC-J which was the primary objective of this research. The results of the analysis which met the inclusion criteria showed that the rate of maternal and fetal complications compared to other reported data from different countries were significantly less [16,17]. The justification for this is that our sample size is consequentially small. Moreover, our population is different in means of demographic features. Overall, emergency CSs were 64.8% and 35.2% for the elective CSs (Table 2). Thus, we found that the association between the rate of maternal complications and the health status of the mother, demographical factors such as age, nationality and body mass index is insignificant, as it was 7% of our sample size. Maternal complications overall were 6.8%. Bleeding and wound extension were the frequent maternal complications as they both accounted for 2.1%. Uterine atony and urinary tract Injury showed 1.1% and 0.7%, respectively. As for the other complications, the estimation was 0.7% (Figure 1). Also, we established if the rate of maternal complications was influenced by the type of CS. It revealed that the elective CS was estimated at 7.1% which is slightly more than the emergency CS reported at 6.6% (Table 4). Furthermore, the association of maternal complications and whether the mother was primigravida or multigravida has been conducted. It was shown that there was also no significance. However, there was a slight increase with the multigravida mothers which was 7% and the primigravida mothers’ was 5.9% (Table 3). In addition, the overall percentage of the pregnant women with comorbidities in our sample size was 40.2% (Table 1). Gestational diabetes and diabetes mellitus reported the most common health condition in our population with 24.6%, as it also showed no significance whatsoever alongside with the number of past CSs due to the fact most of them had their disease under control either by diet or insulin. Hypothyroidism, which was the second-highest comorbidity, counted for 8.5%. Both hypertension and asthma were estimated at 3.6%. As for preeclampsia, its rate was 2.5%. Both pregnancy cholestasis and anemia conveyed 1.4%. Other comorbidities were approximately 9.6% (Table 1). 

On the other hand, the rate of fetal complications was almost 6% with low APGAR Score and NICU admissions being the most frequent complication at 2.1% for each (Figure 1). Fetal stress and fetal death both accounted for 0.7%. Fetal complications in association with type of CSs reported 8.2% for emergency CSs and 1% for elective CSs. The percentage of pregnant women who received blood transfusion was 7.5%. Additionally, the rate of fetal complications in association with the maternal health status, demographical factors and number of CSs showed no significance as well.

 

**Figure 2:**
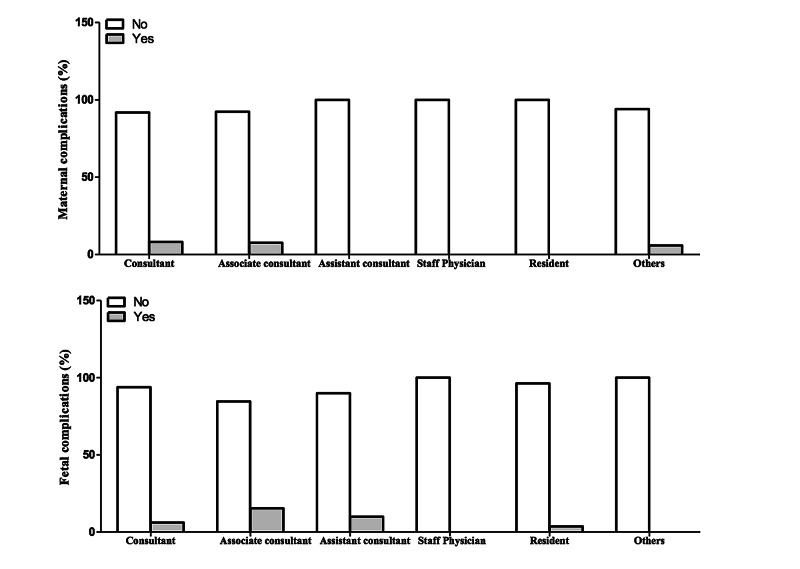
Bar graphs showing associations between operations and complication.

Into the bargain, most CSs were operated by consultants which reported approximately 70%. Residents came second as they gave approximately 10%. Rate of maternal complications and fetal complications for consultants was 8.2% and 6.2%, respectively. As for associate consultants, maternal complications showed 7.7% and fetal complications showed 15.4%. Assistant consultants reported 10% for fetal complications. Residents reported only 3.7% for fetal complications. The justification for the high rate complication for consultants is that they are the most frequent surgeons and complicated cases are handled by them (Table 5, Figure 2). 

 

**Table 5:** Cross-tabulation between fetal and maternal complications and operators.

Operators	Maternal complications		Fetal complications	
	No (n = 262)	Yes (n = 19)	No (n = 265)	Yes (n =16)
Consultant (n =195)	179 (91.8%)	16 (8.2%)	183 (93.8%)	12 (6.2%)
Associate Consultant (n =13)	12 (92.3%)	1 (7.7%)	11 (84.6%)	2 (15.4%)
Assistant Consultant (n = 10)	10 (100.0%)	-	9 (90.0%)	1 (10.0%)
Staff Physician (n = 2)	2 (100.0%)	-	2 (100.0%)	-
Resident (n = 27)	27 (100.0%)	-	26 (96.3%)	1 (3.7%)
Others (n = 34)	32 (94.1%)	2 (5.9%)	34 (100.0%)	-

## Conclusions

This literature review found evidence that the most common maternal complications in women who underwent CS in 2017 at KAMC-J accounted for 6.8%, as bleeding and wound extension were the most frequent ones as 2.1% each. Fetal complications were noticed as well and accounted for 5.7%, as low APGAR score and NICU admissions were the most frequent ones. Maternal and fetal complications in association with any of the factors discussed showed no significance. As for the secondary objective, the association between operator level and rate of complications revealed the highest percentage of complications associated with consultants mainly because the number and complications of the cases were higher compared to residents and others. Additionally, performance of residents who worked in KAMC-J in 2017 provides evidence of safe practice. More opportunities to perform CS by residents is suggested. 
